# Mechanism of the pH-Induced Conformational Change in the Sensor Domain of the DraK Histidine Kinase via the E83, E105, and E107 Residues

**DOI:** 10.1371/journal.pone.0107168

**Published:** 2014-09-09

**Authors:** Kwon Joo Yeo, Young-Soo Hong, Jun-Goo Jee, Jae Kyoung Lee, Hyo Jeong Kim, Jin-Wan Park, Eun-Hee Kim, Eunha Hwang, Sang-Yoon Kim, Eun-Gyeong Lee, Ohsuk Kwon, Hae-Kap Cheong

**Affiliations:** 1 Division of Magnetic Resonance, Korea Basic Science Institute (KBSI), Ochang, Chungbuk, Republic of Korea; 2 Chemical Biology Research Center, Korea Research Institute of Bioscience and Biotechnology (KRIBB), Ochang, Chungbuk, Republic of Korea; 3 College of Pharmacy, Kyungpook National University, Kyungpook, Republic of Korea; 4 Biochemicals and Synthetic Biology Research Center, KRIBB, Yuseong-Gu, Daejeon, Republic of Korea; MRC National Institute for Medical Research, United Kingdom

## Abstract

The DraR/DraK two-component system was found to be involved in the differential regulation of antibiotic biosynthesis in a medium-dependent manner; however, its function and signaling and sensing mechanisms remain unclear. Here, we describe the solution structure of the extracellular sensor domain of DraK and suggest a mechanism for the pH-dependent conformational change of the protein. The structure contains a mixed alpha-beta fold, adopting a fold similar to the ubiquitous sensor domain of histidine kinase. A biophysical study demonstrates that the E83, E105, and E107 residues have abnormally high pKa values and that they drive the pH-dependent conformational change for the extracellular sensor domain of DraK. We found that a triple mutant (E83L/E105L/E107A) is pH independent and mimics the low pH structure. An i*n vivo* study showed that DraK is essential for the recovery of the pH of *Streptomyces coelicolor* growth medium after acid shock. Our findings suggest that the DraR/DraK two-component system plays an important role in the pH regulation of *S. coelicolor* growth medium. This study provides a foundation for the regulation and the production of secondary metabolites in *Streptomyces*.

## Introduction

Bacteria of the genus *Streptomyces* are one of the most important groups of microorganisms for the production of a large variety of valuable secondary metabolites, including antibiotics, anti-tumor agents and immunosuppressants [1], [2]. Streptomycetes are Gram-positive soil bacteria and have genomes with a high GC content and a complex life cycle involving morphological differentiation [3]. *Streptomyces coelicolor*, the best known species of the *Streptomyces* genus, harbors a variety of secondary metabolite biosynthetic gene clusters for multiple antibiotics, such as blue-pigmented polyketide actinorhodin (ACT), red-pigmented undecylprodigiosin (RED), calcium-dependent antibiotic (CDA), and yellow-pigmented type I polyketide (yCPK) [1], [4]–[6].

Regulation of secondary metabolite biosynthesis involves complex interactions of pathway-specific and global regulators that activate or repress the expression of corresponding biosynthetic genes depending on various conditions. In the case of global regulators, many are members of the two-component system (TCS), which is the predominant signal transduction system employed by bacteria to sense and respond to environmental changes [7], [8]. The TCS consists of a membrane-embedded histidine kinase (HK), containing an N-terminal sensor (or input) domain and a conserved cytoplasmic kinase domain, and its cognate response regulator (RR), containing an N-terminal receiver domain and a C-terminal output domain. Upon sensing external stimuli, the sensor domain of HK undergoes a conformational change resulting in phosphorylation of a conserved histidine in its cytoplasmic kinase domain. The phosphorylated HK transfers its phosphoryl group to a specific aspartate residue on the cognate RR. The phosphorylated RR modulates the expression of target genes through protein-DNA and protein-protein interactions to sense and adapt to the stimuli [9], [10].

From genomic analysis of *S. coelicolor*, 84 HKs were detected, and 68 were paired with a RR [11]. However, only a few TCSs in this organism have known functions to date [12]–[22]. For example, the VanR/VanS TCS in *S. coelicolor* is known to sense vancomycin through direct interaction with VanS and confer vancomycin resistance through activation of the expression of the *vanH*, *vanA* and *vanX* genes by phosphorylated VanR [22]. The PhoP/PhoR TCS, which mediates a cellular response to phosphate starvation, has also been characterized in *S. coelicolor* [15]. More recently, it was reported that the DraR/DraK TCS of *S. coelicolor* is involved in the differential regulation of antibiotic biosynthesis in minimal medium containing high concentrations of nitrogen sources, such as Glu, Gln, Thr, KNO_3_, and (NH_4_)_2_SO_4_ [21]. Activation of the DraK HK leads to autophosphorylation of its kinase domain in the cytoplasm and subsequent transphosphorylation of DraR, activating *act*II-ORF4 gene expression to promote ACT production.

Our previous study has revealed that the extracellular sensor domain (ESD) of DraK undergoes a reversible, pH-dependent conformational change in the pH range of 2.5–10 [23]. Analysis of CD and NMR data suggested that the ESD of DraK has a more structured form (MSF) below pH 5.0 and a less structured form (LSF) above pH 7.5. Moreover, when acidic-pH shock was applied to surface-grown *S. coelicolor* cultures, ACT production and the expression of an *act*II-ORF4 gene pathway-specific regulator were also highly enhanced [24]. From this point of view, it is conceivable that DraK is involved in the pH regulation of *S. coelicolor*.

To understand the mechanism of the pH-dependent conformational change in the ESD of DraK, we solved the solution structure of the ESD and examined structural properties of various mutants based on the pKa values of glutamate residues. In addition, to understand the pH regulation by the DraK/DraR TCS, we investigated changes in the pH profiles of culture media and phenotypes of *draR* or *draK*- deleted *S. coelicolor* mutants after acidic-pH shock.

## Materials and Methods

### Cloning, expression, and purification of the ESD

The DNA sequence corresponding to the ESD of DraK (residues E28-R115) was amplified by polymerase chain reaction (PCR) from the genomic DNA of *S. coelicolor* A3(2). Following digestion with the *Bam*HI and *Xho*I restriction enzymes, the purified PCR product was cloned into the pGEX-4T-1 vector using T4 ligase, resulting in pGST (glutathione S-transferase-binding protein)-ESD. *Escherichia coli* BL21 transformants harboring GST-ESD was incubated for 20 h at 20°C after induction by 0.5 mM isopropyl β-D-thiogalactopyranoside (IPTG). The purification process was performed following the previous report by [23]. For ^15^N-labeled or ^15^N and ^13^C-labeled proteins, cells were grown in M9 minimal medium supplemented with ^15^NH_4_Cl or ^15^NH_4_Cl and ^13^C-labeled D-glucose, respectively. To produce the mutant ESD used in this study, pGST-ESD was mutated with the QuikChange Site-Directed Mutagenesis Kit (Stratagene).

### NMR spectroscopy

NMR measurements were performed at 25°C with Avance 500 and Avance II 900 spectrometers (Bruker). A total of 1 mM ^15^N- and ^13^C-labeled DraK ESD (E83Q) was used for 3D structure determination in a buffer containing 10 mM sodium acetate, pH 4.5, 50 mM NaCl, and 10% D_2_O. The ^1^H, ^15^N, and ^13^C resonances of the backbone and side chain were assigned by analyzing 3D heteronuclear correlation spectra: for backbone assignment, HNCO, HNCA, HN(CO)CA, HN(CA)CO, NHCACB, and CBCA(CO)NH [25]–[28], and for side chain assignment, HBHANH, HBHA(CO)NH, CCH-TOCSY, and HCCH-TOCSY [29]–[30]. Distance restraints were obtained from ^15^N- or ^13^C-resolved 3D NOE spectra. ^1^H-^15^N residual dipolar coupling constants for the partially aligned protein in medium with a stretched polyacrylamide gel were obtained from IPAP-HSQC experiments. All NMR spectra were processed with Bruker Topspin 3.0 and analyzed with Sparky 3.113 (Goddard T.G. and Kellner D.G., University of California, San Francisco).

### Structure determination

Structures were calculated by CYANA (version 3.0) [31], and they were coupled with automatic NOESY assignments using the CANDID algorithm [32]. We repeated the CYANA/CANDID runs until obtaining a final result that satisfied geometry-based criteria. A total of 1,750 meaningful NOE upper distance restraints were obtained by CANDID (398 intra-residual, 488 sequential, 390 medium range, and 474 long range). Finally, the 100 structures that showed no significant violation against distance restraints were generated and were further refined with the AMBER package (version 12) [33]. In this stage, in addition to distance restraints, we added 80 backbone H-N derived residual dipolar coupling restraints. RDC data were analyzed using PALES [34]. AMBER refinement consisted of 1,500-step minimization, 40-ps restrained simulated annealing and 1,500-step minimization stages. To approximate solvent effects, we employed the generalized Born implicit solvent model [35]. The 20 structures that showed the lowest AMBER energies were chosen as a final ensemble and analyzed according to wwPDB recommendations [36]. We deposited the coordinates and NMR restraints for the structure calculation in the PDB database (2MJ6) and BMRB database (accession code: 19707).

### Determination of the pKa values for the glutamate side chains of the ESD

The pKa values for the side-chain carboxyl groups of the glutamate side chains were obtained by analyzing the pH titration curves of the carboxyl groups, which are indirectly observed by chemical shifts in their γ carbons adjacent to carbonyl carbon (CO) using 2D ^1^H-^13^C HCCH TOCSY spectra. The spectra were recorded with 0.6 mM protein (wild type) selectively enriched with ^13^C glutamate in a range of pH 2.6–6.4. The pKa values were calculated by a nonlinear least-squares curve fitting of the Henderson-Hasselbalch equation [37]: δ_exp_ = (δ_A_+δ_B_10^(pH-pKa)^)/(1+10^(pH-pKa)^), where δ_A_ and δ_B_ are the plateau values of the γ carbon chemical shifts in the acidic and basic pH limits, respectively.

### Circular dichroism (CD) spectroscopy

CD experiments were performed using a J-710 spectropolarimeter (JASCO) at 25°C with a 1 mm path length cylindrical quartz cell. CD data were obtained from five scans with an average scan rate of 0.2 nm/s. A total of 30 µM protein in 50 mM NaCl was titrated using microliter aliquots of 0.05–0.1 M of HCl or NaOH. The pKa values were calculated from the CD data instead of that from chemical shifts by fitting the above equation.

### Fluorescence spectroscopy

Fluorescence of the tyrosine residue at position 75 in the ESD of DraK was measured by excitation at the 277 nm wavelength using a Cary Eclipse Fluorescence Spectrophotometer (Varian). Fluorescence data were obtained from 10 µM protein containing 50 mM NaCl in a 1 ml quartz cell.

### Construction of the *draR* and *draK* gene deletion mutants in *S. coelicolor*

All gene disruptions were performed according as previously reported using the pKC1139 plasmid to deliver corresponding kanamycin resistance gene disruption cassettes [38] (Figure S1 in Appendix S1). A 1.1-kb DNA fragment from pFDneo-S encoding the *aphII* gene responsible for kanamycin resistance was routinely used as a selective marker for the construction of the gene disruption cassette. These gene disruption cassettes consisted of two PCR-derived DNA fragments, which correspond to the flanking regions of the target gene and are bridged by the kanamycin resistance cassette. Detailed primer information for each construct is summarized in Table S1 in Appendix S1. The gene disruption plasmids pKC-3063A and pKC-3062B were delivered into *S. coelicolor* A3(2) cells by conjugation with *E. coli* ET12567(pUZ8002) (Figure S1 in Appendix S1). Intergeneric conjugation between *E. coli* and *Streptomyces* was performed as previously described with minor modifications. Transformants resistant to both apramycin and kanamycin were selected and grown in fresh R2YE/kanamycin liquid medium at 37°C for 4 days to force the integration of disruption cassette DNA from gene disruption vectors into chromosomal DNA. The resulting gene disruption mutants were selected on R2YE/kanamycin medium and confirmed by PCR with relevant primer sets (Table S1 in Appendix S1) using total genomic DNA from each mutant as template (Figure S2 in Appendix S1).

### pH profile after acidic pH shock cultivation of the *draR* and *draK* gene deletion mutants

pH shock experiments and pH profiling were performed as previously described with minor modifications [24]. *S. coelicolor* A3(2) were grown on a cellophane film placed on supplemented minimal medium (SMM) plates containing 2 g/L casamino acids, 9 g/L glucose, 1 mM NaH_2_PO_4_, 1 mM K_2_HPO_4_, 5 mM MgSO_4_, 20 g/L agar, and trace elements at 28°C. A SMM plate with no TES buffer was used to eliminate the buffering effect, thus allowing pH changes during cultivation. The initial pH was 7.1 after autoclaving. Cells were cultivated for 2 days before being transferred to a new SMM plate with a pH of 4.5. The pH 4.5 SMM plate was adjusted with 0.1 N HCl. Just before transfer, the pHs of the medium were approximately 5.6 (wild type), 6.4 (Δ*draK*), and 5.4 (Δ*draR*). The transferred cells were incubated for an additional 7 days (9 days in total). The pH of the solid medium was measured using Test paper (ToyoRoshi Kaisha, Japan).

## Results

### Overall structure and comparison of the DraK ESD structure with the sensor domain of CitA

**Figure 1:**
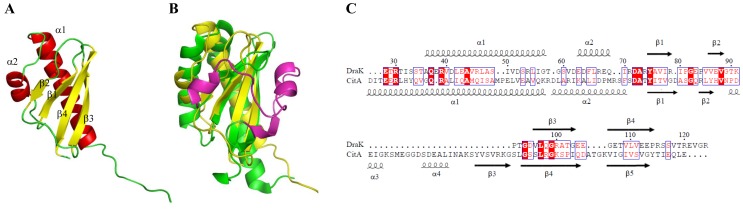
Solution structure of the ESD (E83Q) and sequence and secondary structure alignments with the sensor domain of CitA. (**A**) A solution structure of the ESD (E83Q) from the 20 lowest-energy structures is represented by the ribbon diagram. Α helices and β strands are colored red and yellow, respectively. (**B**) The structure of the ESD (yellow) and that of the CitA sensor domain (green) are superimposed. The magenta color indicates the absent structural elements in the ESD of DraK. (**C**) Sequence alignment and comparison of the secondary structure between the sensor domain of DraK and that of CitA. The red blocks denote regions of sequence identity across the two domains, and the blue boxes indicate partially conserved residues.

We determined the ESD (E83Q) mutant structure at pH 4.5. Although the wild type ESD (residues E28-R115) shows some dimeric form in solution at pH 4.5, the protein exists mainly as monomer [23], enabling structure determination for the MSF at pH 4.5 using heteronuclear NMR spectroscopy. In addition, the ESD (E83Q) mutant provides a significant improvement in spectral quality compared with the wild type. The HSQC spectrum of the E83Q mutant was almost similar except for the mutated region to that of the wild type, indicating that the structure of the E83Q mutant represents that of the wild type (Figure S3 in ). Structural statistics for the ESD (E83Q) are described in Table S2 in Appendix S1. Figure 1A shows the solution structure of an ESD derived from the 20 lowest-energy NMR structures (r.m.s.d. of 0.29 Å and 0.78 Å for the backbone and all heavy atoms, respectively), including a total of 1,750 NOE distance restraints and 82 global orientation restraints from ^1^H-^15^N residual dipolar couplings (RDCs) (Table S2 in Appendix S1). The overall structure of the ESD consists of 2 α-helices and 4 stranded β-sheets. The structure is well ordered in the region (T35-R115), while the first seven residues in the N-terminus (E28-S34) are not ordered. The α1 (residues A36-G57) and α2 (residues E63-Q69) helices are located on the backside of the β-sheet, and the α2 helix connects a pair of antiparallel β-strands, β1 (residues Y75-R79) and β2 (residues V86-V88). The long loop (residues G89-D95) connects the β2 and β3 (residues V96-G103) strands across the ends of the β1 and β4 (residues G106-P114) strands. The β4 strand is located between the β1 and β3 strands, with an antiparallel arrangement.

The ESD of DraK contains a typical mixed alpha-beta fold, which is one of the structural classes of HK sensor domains [39]–[40]. In addition, despite low sequence homology with the sensor domain of CitA, which regulates citrate metabolism in *Klebsiella pneumoniae*, the overall structure of the ESD is similar to that of sensor domain of CitA, and the ESD structure could be superimposed on the sensor domain of CitA with the exception of the region from α3 to β3 including the loop-helix-loop motif located in front of the β-sheet of CitA (Figure 1B). Secondary structural alignment with the sensor domain of CitA (PDB code: 2J80) indicated that the loop-helix-loop motif (between β2 and α4), which is a highly conserved structure in PAS (PER-period circadian protein, ARNT-aryl hydrocarbon receptor nuclear tranlocator protein, SIM-single-minded protein) folds of HKs [40]–[42] and serves as a citrate binding pocket, is absent in the ESD of DraK (Figure 1C). Furthermore, instead of 5 β-strands, only 4 β-strands are located between the N- and C-terminal helices in the ESD of DraK. The β3 and β4 strands of the ESD correspond to the β4 and β5 strands of the CitA, respectively, because of the lack of the structural elements (α3 to β3) of CitA in the ESD of DraK (Figure 1C). The last 9 residues which are predicted as extracellular domain were easily degraded during purification and NMR experiment. In case of CitA, the corresponding C-terminal residues also exhibit unstructured form. There is no remarkable sequence homology between the C-terminus of the sensor domain of CitA and that of the ESD of DraK (Figure 1C).

In general, HKs employing a PAS fold for signal sensing have sensor domains comprising more than 120 amino acids to form a PAS core on the β-sheet fold, whereas the ESD of DraK comprising 97 amino acids (residues E28-R124) appears to not be sufficiently long enough to form a PAS core on the β-sheet of the ESD of DraK. The absence of a PAS fold in the ESD might suggest a different signal recognition mechanism compared with HKs sensing a signal molecule through the PAS fold.

### The ESD is not completely unstructured at pH 7.5, and the pH-dependent conformational change alters the conformation of its C-terminus

**Figure 2:**
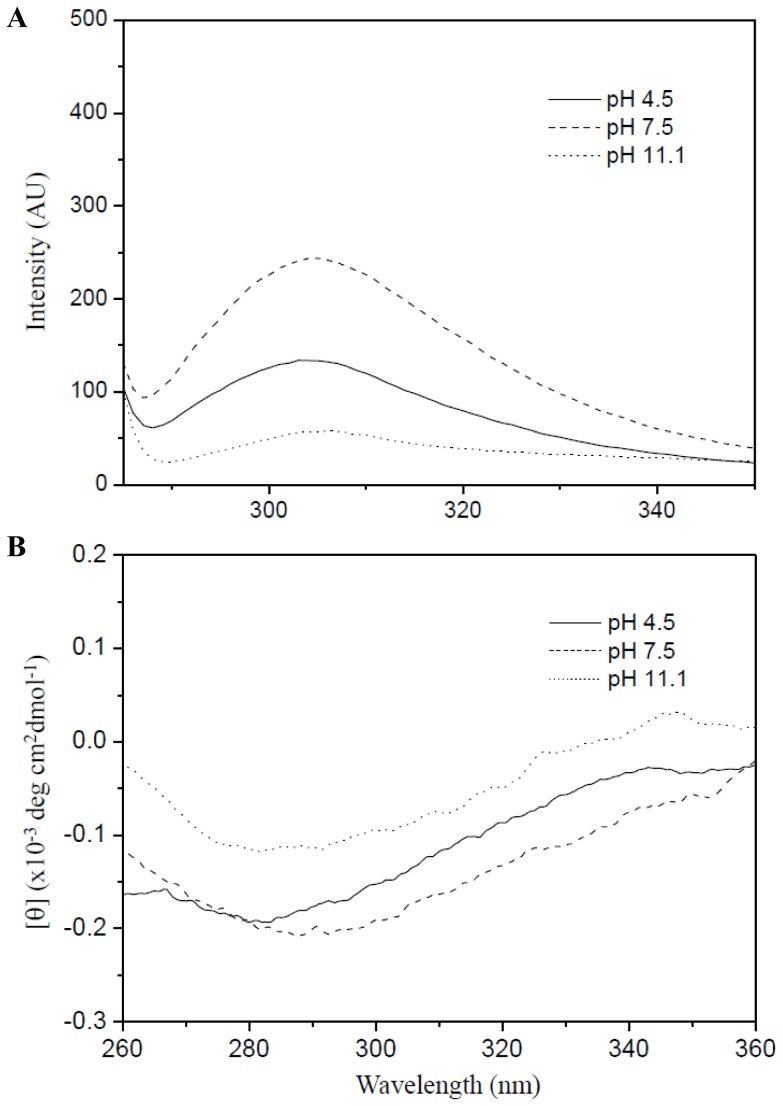
pH-dependent fluorescence and near-UV CD spectra of the ESD. The signals were measured at pH 4.5 (solid line), 7.5 (dashed line), and 10.0 (dotted line). The excitation wavelength was 277 nm. (**A**) and (**B**) denote fluorescence and near-UV CD spectra, respectively.

In a previous report, we observed that the ESD has a reversible conformational change in the pH range of 2.5-10 with a major transition at pH 6.1, and it was more structured at pH 4.5 but less structured at pH 7.5 [23]. In addition, the previous CD result indicated that some α-helical structure is still remained in the ESD at pH 7.5 [23]. To confirm that the ESD exists in structured form at pH 7.5 and to monitor the conformational change of the C-terminus of the ESD by pH alteration, we compared fluorescence and near-UV CD spectra for the ESD at three different pH values (pH 4.5, 7.5, and 10.5). In general, fluorescence from tyrosine side chains within a protein is quenched in an unfolded state because the tyrosine side chain is exposed to solvent in the unfolded state. There is a tyrosine at position 75 that is closely positioned near the C-terminus of the ESD. As shown in Figure 2A, a maximum peak appears at around 305 nm, and the signal intensity is significantly changed at each pH value. The signal intensity is increased at pH 7.5 compared with pH 4.5, indicating that the side chain environment of the Y75 residue at pH 7.5 becomes more hydrophobic than that at pH 4.5. In other words, the Y75 side chain is not completely exposed to solvent at pH 7.5. In contrast, the signal intensity of fluorescence at pH 11.1 was significantly decreased, indicating that the Y75 side chain could be anionic state and exposed to solvent because the pKa value of the side chain of tyrosine is approximately 10.1. Probably, the protein becomes unstructured (or aggregated) at that pH. In addition, the near-UV CD spectra show that the amplitude of the negative band in the near-UV at pH 7.5 is increased compared with pH 4.5, reflecting that the environment of the Y75 residue at pH 7.5 is more rigid and more structured (Figure 2B). The signal intensity of near-UV CD is highly decreased at pH 11.1, suggesting that the protein becomes unstructured (or aggregated) at that pH. The results from near-UV CD spectra are consistent with those from fluorescence spectra. Thus, together with the previous CD results [23], the fluorescence and near-UV CD spectra suggest that the protein remains structured around the Y75 residue at pH 7.5 despite being less structured overall. These data also suggest that the pH-dependent conformational change of the ESD induces a conformational change of its C-terminus because Y75 is close to the C-terminus. The conformational change of the C-terminus of sensor domain of HK is important for transferring its external signal to its transmembrane domain.

### The pKa values of carboxyl groups of glutamate side chains of the ESD

In general, the pH-dependent protein conformational change around pH 6.0 occurs due to the protonation or deprotonation of histidine side chain and is important for protein activity and stability. However, the ESD of DraK does not contain histidine residues. Frequently, acidic amino acids (glutamate and aspartate) play an important role in protein stability and enzyme activity through alteration of their pKa values [43]–[45]. Therefore, the pH-dependent conformational change of the ESD could be influenced by acidic amino acids. In the solution structure, with the exception of the E107 residue, the carboxyl groups of all of the glutamate and aspartate side chains are exposed to solvent, which otherwise form salt bridges with arginine side chains. In contrast, the carboxyl group of the E107 residue is buried in the structure, suggesting that the carboxyl group may be protonated at pH 4.5 and has abnormally high pKa value. Moreover, in our previous study, we found that the E83 residue was critical for the pH-dependent conformational change of the ESD [23]. Thus, E83 and other glutamates may be involved in the pH-dependent conformational change of the ESD. To understand the pH-dependent conformational change of the ESD in detail, we attempted to determine the pKa values of all glutamate residues for the wild type ESD.

**Figure 3:**
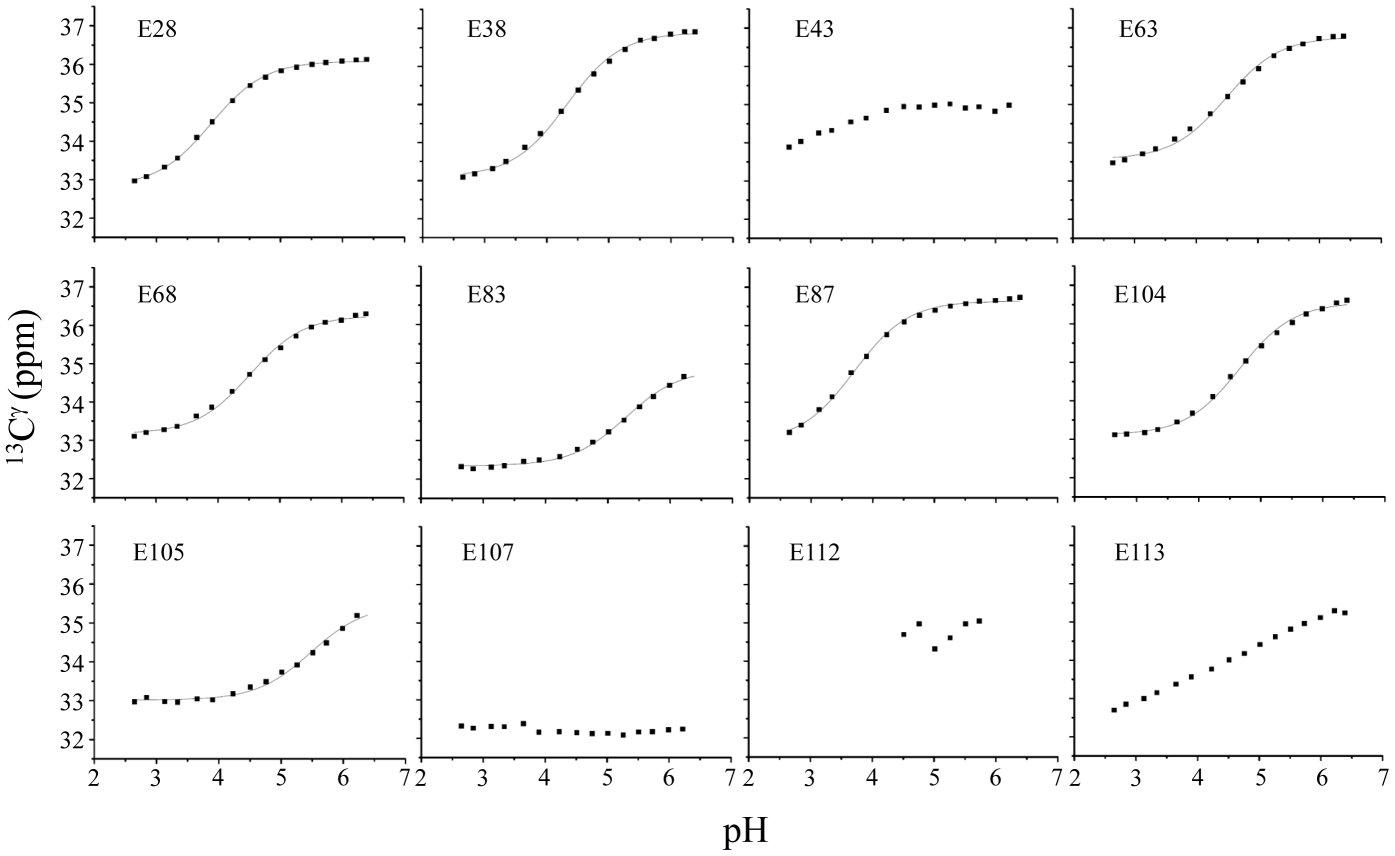
The pH-dependent γ-^13^C chemical shifts for all of the glutamate residues in the ESD. The pKa values were determined by fitting the data using the Henderson-Hasselbach equation (solid line). The pKa values are listed in Table 1.

NMR spectroscopy is a powerful method for determining pKa values for ionizable amino acids from pH-dependent chemical shift variations. We attempted to calculate pKa values from variations in the chemical shifts of the γ carbons of all glutamate residues in the ESD as a function of pH (Materials and Methods). We were able to determine the pKa values for 8 of 12 glutamate residues by NMR spectra, excluding E43, E107, E112, and E113. The titration data were recorded up to pH 6.4 because the signal intensities of the γ carbons of all of the glutamate residues were highly decreased due to the low population of the structured form above pH 6.4. The pH-dependent chemical shifts in the γ carbon resonances for all of the glutamate residues in the ESD are shown in Figure 3. The pKa values determined by nonlinear least-squares curve fitting using the Henderson-Hasselbalch equation are listed in Table 1. As shown in Figure 3, the E83 and E105 residues showed significant shifts from their normal pKa values. Most of the titration curves show simple sigmoidal shapes between the ionized (deprotonated) and neutral (protonated) states of the carboxyl group, with the exception of E43, E107, E112 and E113. The γ carbon resonances for most glutamate residues appear to be approximately 36–37 ppm in the deprotonated state and approximately 32–33 ppm in the protonated state. E112 γ carbon resonance was not observed above pH 5.8, and the changes in the chemical shifts of the E43, E107 and E113 residues had a linear but not sigmoidal shape. Thus, the pKa values for E43, E107, E112, and E113 could not be determined using this technique. Although the carboxyl group of the E107 residue is not titratable, the E107 γ carbon resonates at approximately 32.5 ppm in the pH range, indicating that E107 is in the protonated state at pH 6.4. Thus, its pKa value is expected to be above 6.4. This result is also compatible with a buried form of the carboxyl group in the structure, reflecting the fact that the carboxyl group of the residue is in a neutral rather than anionic state.

**Table 1:** pKa values of the glutamate residue side chains in the ESD.

E28	3.9
E38	4.3
E43	-
E63	4.5
E68	4.5
E83	5.3
E87	3.7
E104	4.6
E105	5.5
E107	8.1*
E112	-
E113	-

*Determined using CD spectra.

**Figure 4:**
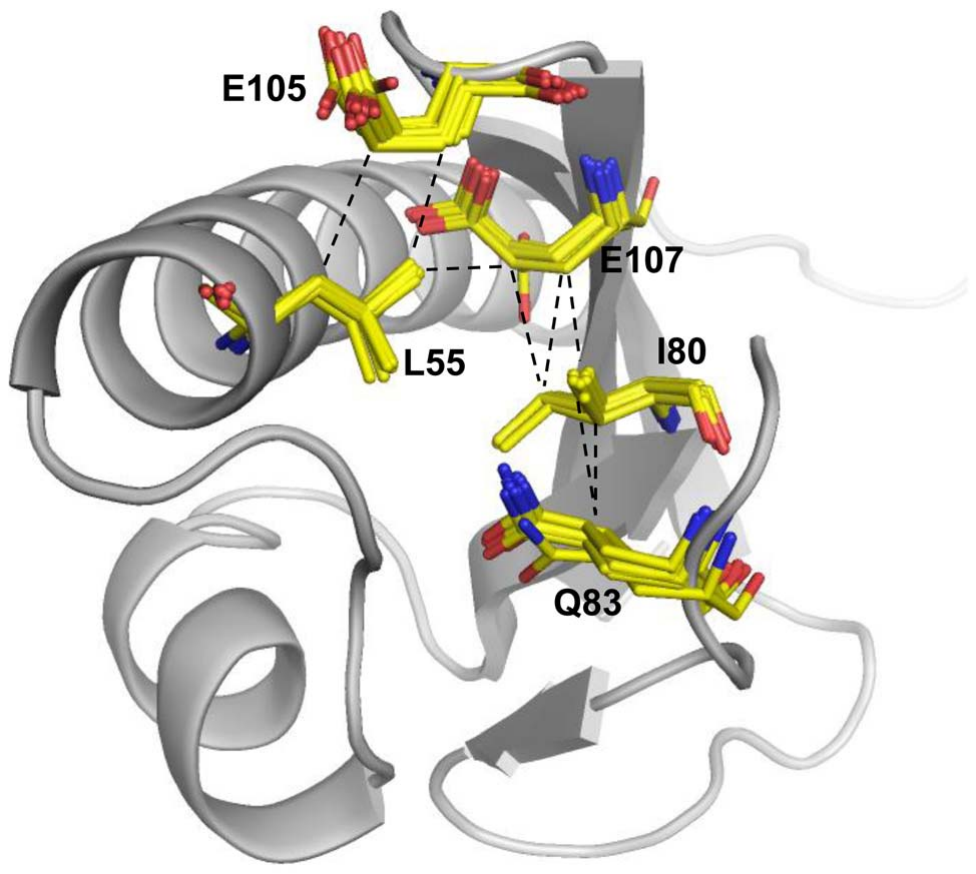
Solution structure of the ESD (E83Q). The Q83, E105, and E107 residues interact with the L55 and I80 residues in the ensemble of 10 lowest-energy structures, which are superimposed with yellow sticks. The dashed line indicates that distances between two carbons are less than 4 angstroms in all of the 10 lowest structures.

Despite abnormally high pKa values for E83 and E105 in the wild type protein, both termini of the Q83 and E105 side chains in the E83Q mutant are exposed to solvent. The hydrocarbon side chains of Q83 and E105 are distinctively stabilized by hydrophobic contacts with the I80 side chain and that of L55, respectively (Figure 4). These results suggest that the neutralization and hydrophobicity of the E83, E105, and E107 residue side chains could generate a pH-dependent conformational change in the protein. To address this possibility, a mutational study of the three glutamate positions was subsequently performed.

### Mutation at position E83

**Figure 5:**
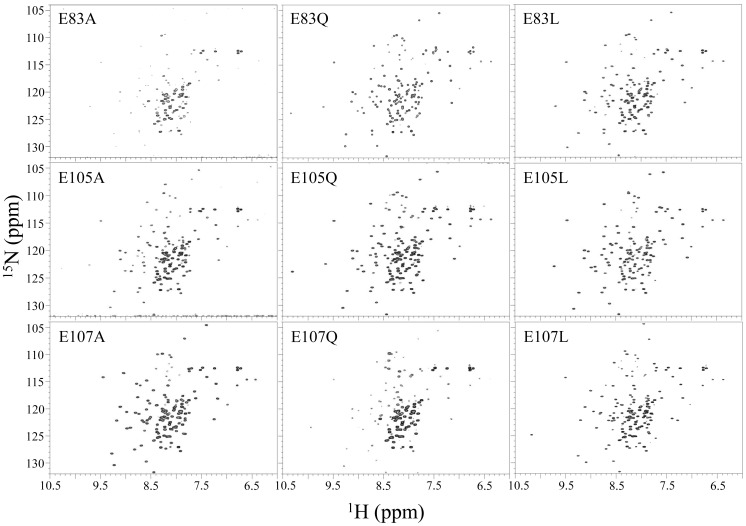
^1^H-^15^N HSQC spectra of the ESD mutants in 10 mM HEPES pH 7.5 and 100 mM NaCl. Each glutamate residue at positions 83, 105, or 107 is mutated to alanine, glutamine, or leucine. ^1^H-^15^N HSQC spectrum of the wild type protein under the same conditions shown in the previous report [23].

In our previous study, we observed that the E83Q mutant shifts the transition point of the pH-dependent conformational change approximately by 1 pH unit compared with the wild type [23]. To verify the influence of the three glutamate residues on the pH-dependent conformational change of the ESD, several mutants (glutamate to glutamine, alanine, and leucine) were characterized using ^1^H-^15^N HQSC spectra. Glutamine mimics the side chain protonation state of the glutamate carboxyl group, alanine removes the charge effect of glutamate, and leucine is more hydrophobic than glutamine and alanine. Figure 5 shows the ^1^H-^15^N HSQC spectra for proteins mutated at position 83. Compared with the wild type protein [23], the mutants favor a MSF than the wild type protein at pH 7.5, suggesting that the neutralization and hydrophobicity of the glutamate residues induce the MSF in high pH solution. Interestingly, the E83Q and E83L mutants demonstrate that approximately 50% of the MSF exists at pH 7.5, whereas only a small amount of the MSF of E83A is formed at pH 7.5 (Figure 5). In particular, the E83D mutant shifts the transition point of the pH-dependent conformational change approximately by 0.4 pH unit lower compared with the wild type (Figure S4 in Appendix S1) [23]. Thus, the length of glutamine hydrocarbon side chain at position 83 is important for the MSF due to its providing a hydrophobic interaction with the I80 side chain (Figure 4). In addition, the E83 negative charge at pH 7.5 appears to interfere with hydrophobic interactions between the E83 and I80 hydrocarbon side chains because the MSF of the wild type protein does not exist at that pH. These results suggest that neutralization, hydrophobicity, and the side chain length at position 83 play an important role in inducing the MSF.

### Mutation at position E105

Similar to E83, the E105 carboxyl group is not buried in the structure (Figure 4); nevertheless, the pKa value of E105 is abnormally high (Table 1). To confirm the effects of neutralization and hydrophobicity at position 105, the E105 residue was mutated to glutamine, alanine, or leucine similar to that for the E83 mutation. As shown in Figure 5, MSFs were observed at pH 7.5 for the mutants at position 105, indicating that neutralization and hydrophobicity at this position induce the MSF at that pH. Both E105Q and E105L have similar effects on the formation of the MSF at pH 7.5 through interaction with the L55 side chain (Figure 4). In contrast, the E105A mutant shows a less amount of MSF compared with E105Q and E105L mutants, suggesting that neutralization and hydrophobicity with a large enough hydrocarbon side chain length at this position are also important to form the MSF at pH 7.5.

### Mutation at position E107

In Figure 4, the E107 side chain residue is fully buried in the structure, suggesting that the E107 carboxyl group may be not in an anionic state at pH 4.5. In addition, the chemical shift value of the E107 γ carbon is approximately 32.5 ppm until pH 6.5. These results suggest that the E107 carboxyl group is protonated at pH 6.5. Therefore, the pKa value for E107 is expected to be a higher than 6.5. As expected from previous results, all three mutants, E107A, E107Q, and E107L, induce the MSF at pH 7.5 (Figure 5), indicating that hydrophobicity at position 107 is also important for the MSF at high pH. Distinctively, the MSF population of the E107A mutant is higher than that for the E107Q mutant. It appears that the short methyl group of alanine is more favorable than the hydrophilic side chain of glutamine because the E107 side chain is fully buried in the hydrophobic space comprising the side chains of the L55 and I80 residues (Figure 4). These results suggest that hydrophobicity is more important than the length of the side chain at position E107, which is unlike the E83 and E105 mutants.

### E83L/E105L double mutation

**Figure 6:**
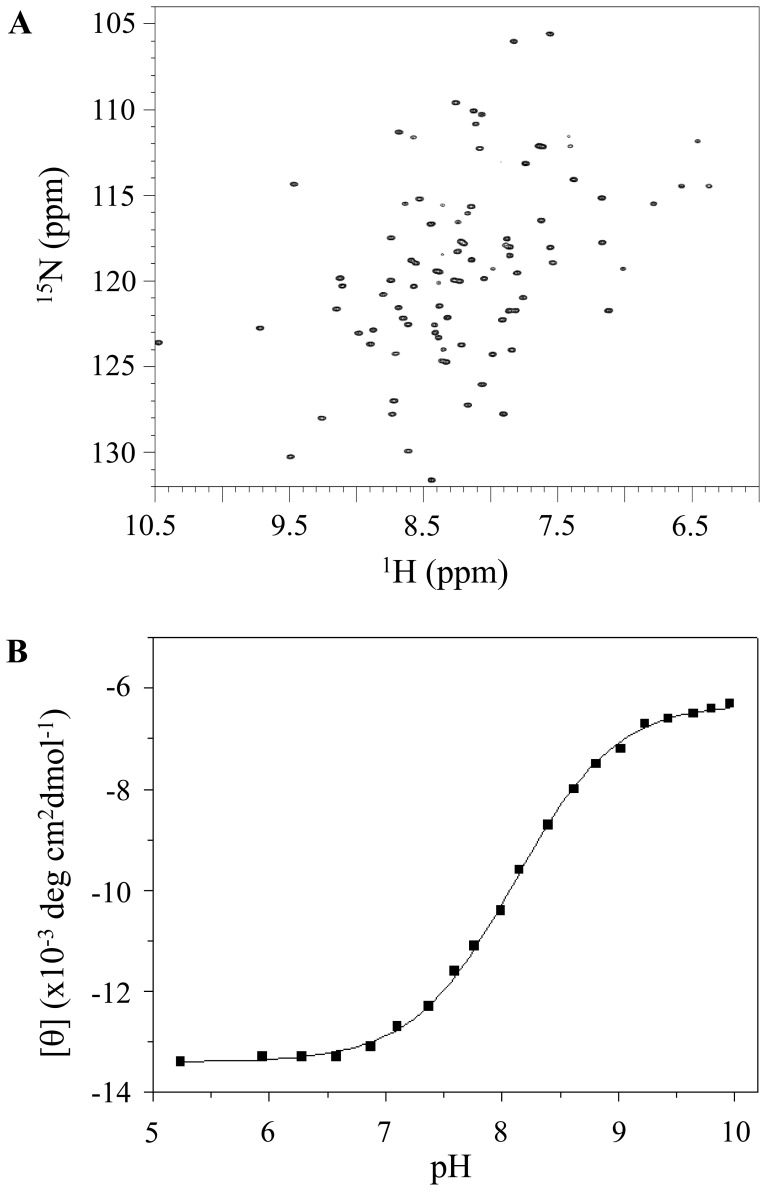
^1^H-^15^N HSQC spectra and the change in the CD signal intensity at 218 nm over a pH range of 5.2-10.0. (**A**) ^1^H-^15^N HSQC spectra for the ESD (E83L/E105) mutant in 10 mM HEPES, pH 7.5 and 10 mM sodium acetate, pH 4.5 containing 100 mM NaCl. (**B**) The pKa value was determined by fitting the data using the Henderson-Hasselbach equation (solid line).

From the above results, we found that the three glutamates that have abnormally high pKa values participate in the pH-dependent conformational change in the ESD. If the pH-dependent conformational change in the ESD only depends on these three glutamates (E83, E105, and E107), the pKa value of E107 could be determined from the E83L/E105L double mutant. According to a mutational study, to induce the MSF, the amino acids at positions 83 and 105 should have a sufficient length of hydrocarbon side chains and be more hydrophobic. Thus, we substituted E83 and E107 with leucine residues. As shown in Figure 6A, this double mutant is fully structured at pH 7.5, indicating that the effects of the E83 and E105 side chains are completely eliminated by the mutations and that the E107 residue does not affect the MSF at pH 7.5. The E107 side chain is conceivably still protonated at that pH. If the pKa value of E107 is higher than 7.5, the pH-dependent conformational change of the mutant could occur above pH 7.5. CD spectroscopy was used instead of NMR spectroscopy to calculate the pKa value for E107 from the E83L/E105L double mutant because the backbone and side chain assignments for the E107 residue in the mutant are required to generate a titration curve using NMR above pH 7.5. As expected, the CD spectra showed that the pH-dependent conformational change in the double mutant occurred above pH 7.5, and the transition point was observed at pH 8.1 (Figure 6B). This result suggests that the pH-dependent conformational change of the double mutant was derived from the E107 residue because the pKa value of the E107 residue was expected to be >6.5 as shown in Figure 3. Therefore, under the assumption that the conformational change of the double mutant only depends on E107 residue, the pKa value of E107 was determined to be 8.1 from the Henderson-Hasselbach equation using CD data instead of chemical shifts. Although the pKa value for the E107 of the E83L/E105L double mutant may differ from the wild type protein because the mutated leucine residues could influence the MSF and LSF states, this result suggests that the E107 residue has a high pKa value (at least above 7.5). In our previous report, a major transition at pH 6.1 and a minor transition at approximately pH 7.5 were observed from CD spectra, but it was unclear whether the signal changes for the minor transition was from the conformational change or experimental error [23]. On the basis of the individual pKa values of all of the carboxyl groups of the ESD, a possible interpretation is that the major transition at approximately pH 6.1 derives from the conformational change by protonation of the carboxyl groups of the E83 and E105 residues because their pKa values are 5.3 and 5.5, respectively, and the minor transition at approximately pH 7.5 occurs via the conformational change by protonation of the E107 carboxyl group because the pKa value of E107 was determined to be 8.1 using the double mutant. Therefore, the E107 pKa value determined by the double mutant using CD spectroscopy is reasonable.

### pH-independent structure of the ESD

If the pH-dependent conformational change in the ESD depends on the three glutamate residues, an ESD triple mutant (E83L, E105L, and E107A) should not undergo a pH-dependent conformational change with varying pH. To address this hypothesis, we generated a triple mutant and monitored conformational changes at various pH values using NMR and CD spectra. ^1^H-^15^N HSQC spectra showed that the protein was fully structured at pH 4.5 and 7.5 (Figure S5 in Appendix S1). From CD spectra, a spectral change was not observed up to pH 10 (Figure S6 in Appendix S1), indicating that a pH-dependent conformational change of the triple mutant does not occur, and the protein is fully structured up to pH 10. Moreover, this result indicates that the pH-dependent conformational change of the double mutant above pH 7.5 derives from deprotonation of the E107 residue because the pH-dependent conformational change between pH 7.5–10.0 disappears in the triple mutant. Finally, our results demonstrate that the pH-dependent conformational change of the ESD derives from only the E83, E105, and E107 residues.

### The DraR/DraK TCS in *S. coelicolor* restores the pH of the growth medium after acid shock

**Figure 7:**
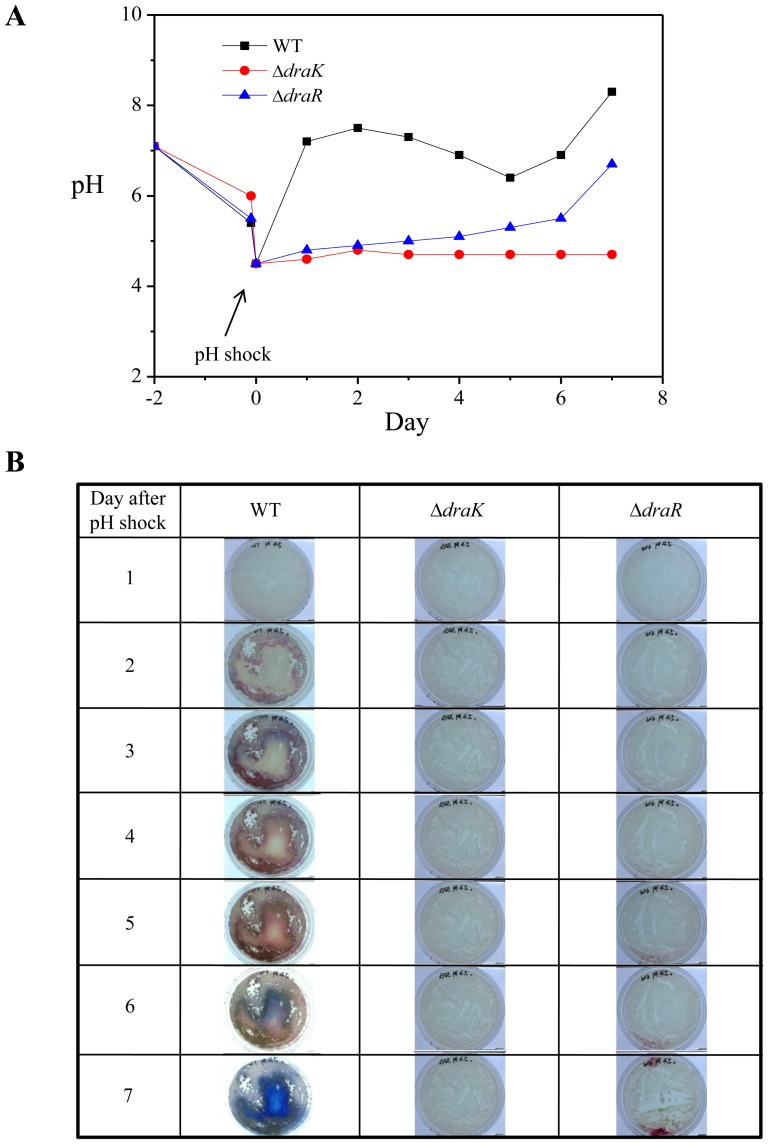
pH profiles and cell phenotypes. (**A**) Time-dependent pH profiles of culture plates for wild type DraK (black), the Δ*draK* mutant (red), and the Δ*draR* mutant (blue) following pH shock. (**B**) Time-dependent cell phenotypes of *S. coelicolor* for wild type DraK, the Δ*draK* mutant, and the Δ*draR* mutant in pH shock culture (PSC).

pH changes in bacterial culture have important roles in signal transduction and secondary metabolite production. Kim and collaborators found that *S. coelicolor* produced more secondary metabolites when allowed to recover pH in the culture medium after a spontaneous or artificial pH drop than when pH changes were suppressed using a buffer [24]. These authors have reported that acidic pH shock enhances the production of the major secondary metabolite ACT in *S. coelicolor*. It has also been reported that ACT production and secretion in *S. coelicolor* are highly enhanced by activation of the DraR/DraK TCS [21]. Moreover, our data show that the conformational change in the ESD of DraK is highly pH sensitive. Based on these results, it is conceivable that the DraR/DraK TCS is related to pH regulation in *S. coelicolor*. To address this hypothesis, we monitored the changes in the pH profile of the culture media and the phenotypes of the *S. coelicolor draR* and *draK* mutants after acidic shock according to previously reported methods [24]. Figure 7 shows the pH profiles and images of a pH-shock culture (PSC). For the wild type protein, the pH dramatically increased soon after the pH shock to reach the neutral level within 2 days and eventually a slightly alkaline level of 8, which is similar to a previous report [24]. However, for the deletion mutant, the pH did not recover to the neutral level. In particular, the *draK* deletion mutant (Δ*draK*) could not recover the pH level at all. In fact, the Δ*draK* mutant was unable to grow after acid shock for a week (Figure 7B). However, when the Δ*draK* mutant was grown for a week in the 4.5 pH shock medium and transferred to fresh pH 7.0 medium, the cells started growing again, indicating that the cells were alive at pH 4.5 (data not shown). In the case of the *draR* mutant (Δ*draR*), it was able to recover the pH even though the recovery pattern was different from that of wild type. The Δ*draR* mutant slowly increased for a week after acid shock, indicating that the mutant maintains the ability to restore the pH of the growth medium although weakly. These results conclusively demonstrated that DraR/DraK in *S. coelicolor* is involved in the regulation of the pH of growth media and morphological differentiation. In particular, the DraK histidine kinase plays a major role in the regulation of the pH of the environment because the Δ*draK* mutant completely loses the ability to recover pH in low-pH growth medium.

## Discussion

Bacterial HKs play an essential role in external stimuli signal transfer by sensing stimuli via the HK sensor domain. Although the function and signaling molecules (or stimuli) of some HKs have been identified, the function of many HKs existing in bacteria remains unknown. In particular, even though the structural change in the sensor domain of HKs is critical for understanding the signal transduction mechanisms of HKs, the structure of many HK sensor domains remains unknown. We had previously shown that the structure of the ESD of DraK exhibits a reversible, pH-dependent conformational change in the pH range of 2.5–10. In particular, the ESD exists as MSF at low pH but it is LSF at high pH. In this study, we solved the solution structure of the ESD of the DraK HK and elucidated the mechanism of its pH-dependent conformational change.

The 3D structure of the ESD of the DraK protein at pH 4.5 could be determined using NMR spectroscopy, whereas it could not be determined at pH 7.5 because of the low stability of the protein and lack of NMR signal at that pH. The overall structure of the protein at pH 4.5 showed a typical HK sensor domain structure containing α/β folds but had no PAS fold. This difference suggests that the ESD of DraK might not serve as a small molecule-binding motif or might possess a different ligand-binding nature compared with the typical sensor domains of HKs because the PAS fold usually provides a binding site for a ligand molecule. From this point of view, the pH-dependent conformational change of the ESD might induce signal transmission through the transmembrane domain and subsequently the cytoplasmic domain of DraK. In fact, fluorescence spectroscopy showed the conformational change of the C-terminal portion of the ESD by a change in pH. The conformational change of the C-terminal portion of the ESD of HK is essential for driving conformational changes of the transmembrane domain.

**Figure 8:**
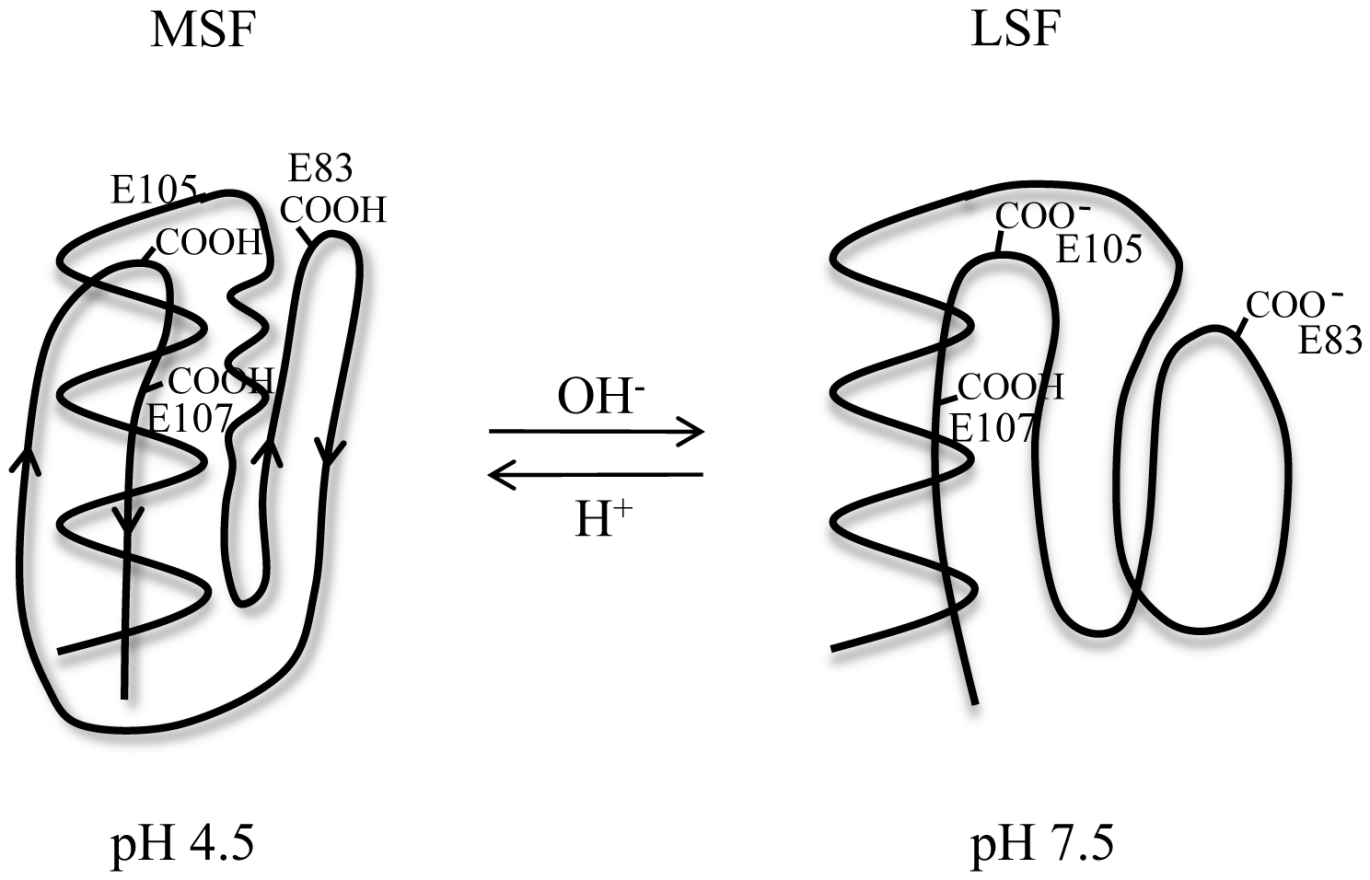
The proposed mechanism of the pH-dependent conformational change of the ESD based on the pKa values of three glutamate residues (E83, E105, and E107) and structural studies. Below pH 5.0, the ESD exists in the MSF, and the three glutamate residues are protonated (left). At pH 7.5, the protein exists in the LSF, and the E107 residue remains protonated (right).

To understand the mechanism of the pH-dependent conformational change of the ESD of DraK in detail, we determined the pKa values of the glutamate residue side chains using NMR and CD spectroscopy. In this report, we found that three glutamate residues (E83, E105, and E107) had abnormally high pKa values and are involved in the pH-dependent conformational change of the ESD of DraK. Based on the pKa values of the glutamate residues, mutational studies allowed us to create a pH-independent triple mutant (E83L, E105L, and E107A) form of the ESD. From the mutational studies of positions E83, E105, and E107, we suggested that protonation of each side chain causes a pH-dependent conformational change, and the neutralization and hydrophobicity of each side chain induces the MSF at pH 7.5. In the case of the E83A mutant, the protein showed a less amount of MSF at pH 7.5 compared with the E105A and E107A mutants. Thus, a longer amino acid side chain at position 83 is more favorable to induce the MSF at pH 7.5. Nevertheless, the alanine methyl is better than the negatively charged glutamate residue in all cases. In addition, the pKa value of the E107 side chain was approximately 8.1, which is the highest value compared with the other two glutamates, suggesting that the E107 side chain is capable of being protonated even under alkali conditions. It is likely that the high hydrophobicity surrounding the E107 residue effectively stabilizes the orientation of protonated side chain of the E107 residue. This high pKa value for E107 may be important for stabilization of the LSF of the DraK ESD between pH 7.5 and 10.0. Our previous report found that the ESD loses the reversible, pH-dependent conformational change ability after exposure to high pHs above pH 11 [23] perhaps because the LSF of the ESD could be destroyed by the deprotonation of E107 above pH 11. As a whole, our results allow us to propose a mechanism for the pH-dependent conformational change of the ESD (Figure 8). Protonation of E83 and E105, having pKa values of 5.3 and 5.5, respectively, drive the reversible pH-dependent conformational changes of the MSF and LSF between pH 4.5 and 7.5, and the protonation of E107, having a high pKa value, maintains the LSF up to pH 10. When the three glutamate residues are fully negatively charged above pH 11, the protein could lose the reversible pH-dependent conformational change ability.

Together with previous studies showing that ACT production and secretion in *S. coelicolor* are highly enhanced by activation of the DraR/DraK TCS [21] and acidic-pH shock [24], our current data showing that the conformational change of the ESD of DraK is highly pH sensitive indicate that the DraR/DraK TCS is involved in the pH-mediated regulation of *S. coelicolor*. To address this assumption, we performed an *in vivo* study using *S. coelicolor* under acid shock growth medium conditions. We found that wild type *S. coelicolor* was able to rapidly restore the pH of the growth medium after acid shock, whereas the Δ*draK* mutant completely lost the pH recovery ability of the growth medium, and the Δ*draR* mutant was able to slowly restore the pH of the medium. Our results demonstrate that the DraR/DraK TCS in *S. coelicolor* is involved in the pH-mediated regulation of the growth medium. Furthermore, DraK appears to play an essential role in growth in acidic pH culture medium, suggesting the possibility that DraK activates not only its cognate RR DraR but also other proteins related to pH regulation. However, to determine whether the pH-dependent conformational change of the ESD by E83, E105, and E107 residues is associated with pH signature or stimulus for the DraK HK, *in vivo* analysis of growth phenotypes and pH recovery of mutant strains expressing DraK with substitutions at E83, E105, and E107 residues, are required.

## Supporting Information

Appendix S1 **Detailed methods and results including NMR and CD spectra.**
(DOCX)

## References

[1] Bentley SD, Chater KF, Cerdeno-Tarraga AM, Challis GL, Thomson NR, et al (2002) Complete genome sequence of the model actinomycete *Streptomyces coelicolor* A3(2). Nature 417: 141–147. 12000953 10.1038/417141a

[2] Challis GL, Hopwood DA (2003) Synergy and contingency as driving forces for the evolution of multiple secondary metabolite production by *Streptomyces* species. Proc Natl Acad Sci U S A 100: 14555–14561. 12970466 10.1073/pnas.1934677100 PMC304118

[3] Hopwood DA (1999) Forty years of genetics with *Streptomyces*: from in vivo through in vitro to in silico. Microbiology 145: 2183–2202. 10517572 10.1099/00221287-145-9-2183

[4] Gramajo HC TE, Bibb MJ (1993) Stationary-phase production of the antibiotic actinorhodin in *Streptomyces coelicolor* A3(2) is transcriptionally regulated. Mol Microbiol 7: 837–845. 7683365 10.1111/j.1365-2958.1993.tb01174.x

[5] Ryding NJ, Anderson TB, Champness WC (2002) Regulation of the *Streptomyces coelicolor* calcium-dependent antibiotic by absA, encoding a cluster-linked two-component system. J Bacteriol 184: 794–805. 11790750 10.1128/JB.184.3.794-805.2002 PMC139508

[6] Takano E, Gramajo HC, Strauch E, Andres N, White J, et al (1992) Transcriptional regulation of the redD transcriptional activator gene accounts for growth-phase-dependent production of the antibiotic undecylprodigiosin in *Streptomyces coelicolor* A3(2). Mol Microbiol 6: 2797–2804. 1435258 10.1111/j.1365-2958.1992.tb01459.x

[7] Stock JB, Ninfa AJ, Stock AM (1989) Protein phosphorylation and regulation of adaptive responses in bacteria. Microbiol Rev 53: 450–490. 2556636 10.1128/mr.53.4.450-490.1989 PMC372749

[8] Perry J, Koteva K, Wright G (2011) Receptor domains of two-component signal transduction systems. Mol Biosyst 7: 1388–1398. 21347487 10.1039/c0mb00329h

[9] Stock AM, Robinson VL, Goudreau PN (2000) Two-component signal transduction. Annu Rev Biochem 69: 183–215. 10966457 10.1146/annurev.biochem.69.1.183

[10] Hoch JA (2000) Two-component and phosphorelay signal transduction. Curr Opin Microbiol 3: 165–170. 10745001 10.1016/s1369-5274(00)00070-9

[11] Hutchings MI, Hoskisson PA, Chandra G, Buttner MJ (2004) Sensing and responding to diverse extracellular signals? Analysis of the sensor kinases and response regulators of *Streptomyces coelicolor* A3(2). Microbiology 150: 2795–2806. 15347739 10.1099/mic.0.27181-0

[12] Yepes A, Rico S, Rodriguez-Garcia A, Santamaria RI, Diaz M (2011) Novel two-component systems implied in antibiotic production in *Streptomyces coelicolor* . PLoS One 6: e19980. 21625497 10.1371/journal.pone.0019980 PMC3098853

[13] Rozas D, Gullon S, Mellado RP (2012) A novel two-component system involved in the transition to secondary metabolism in *Streptomyces coelicolor* . PLoS One 7: e31760. 22347508 10.1371/journal.pone.0031760 PMC3276577

[14] Sola-Landa A, Moura RS, Martin JF (2003) The two-component PhoR-PhoP system controls both primary metabolism and secondary metabolite biosynthesis in *Streptomyces lividans* . Proc Natl Acad Sci U S A 100: 6133–6138. 12730372 10.1073/pnas.0931429100 PMC156338

[15] Sola-Landa A, Rodriguez-Garcia A, Franco-Dominguez E, Martin JF (2005) Binding of PhoP to promoters of phosphate-regulated genes in *Streptomyces coelicolor*: identification of PHO boxes. Mol Microbiol 56: 1373–1385. 15882427 10.1111/j.1365-2958.2005.04631.x

[16] Santos-Beneit F, Rodriguez-Garcia A, Sola-Landa A, Martin JF (2009) Cross-talk between two global regulators in *Streptomyces*: PhoP and AfsR interact in the control of afsS, pstS and phoRP transcription. Mol Microbiol 72: 53–68. 19220751 10.1111/j.1365-2958.2009.06624.x

[17] Shu D, Chen L, Wang W, Yu Z, Ren C, et al (2009) afsQ1-Q2-sigQ is a pleiotropic but conditionally required signal transduction system for both secondary metabolism and morphological development in *Streptomyces coelicolor* . Appl Microbiol Biotechnol 81: 1149–1160. 18949475 10.1007/s00253-008-1738-1

[18] McKenzie NL, Nodwell JR (2007) Phosphorylated AbsA2 negatively regulates antibiotic production in Streptomyces coelicolor through interactions with pathway-specific regulatory gene promoters. J Bacteriol 189: 5284–5292. 17513473 10.1128/JB.00305-07 PMC1951880

[19] Ishizuka H, Horinouchi S, Kieser HM, Hopwood DA, Beppu T (1992) A putative two-component regulatory system involved in secondary metabolism in *Streptomyces* spp. J Bacteriol 174: 7585–7594. 1339426 10.1128/jb.174.23.7585-7594.1992 PMC207469

[20] Lu Y, Wang W, Shu D, Zhang W, Chen L, et al (2007) Characterization of a novel two-component regulatory system involved in the regulation of both actinorhodin and a type I polyketide in *Streptomyces coelicolor* . Appl Microbiol Biotechnol 77: 625–635. 17899070 10.1007/s00253-007-1184-5

[21] Yu Z, Zhu H, Dang F, Zhang W, Qin Z, et al (2012) Differential regulation of antibiotic biosynthesis by DraR-K, a novel two-component system in *Streptomyces coelicolor* . Mol Microbiol 85: 535–556. 22676800 10.1111/j.1365-2958.2012.08126.x

[22] Koteva K, Hong HJ, Wang XD, Nazi I, Hughes D, et al (2010) A vancomycin photoprobe identifies the histidine kinase VanSsc as a vancomycin receptor. Nat Chem Biol 6: 327–329. 20383152 10.1038/nchembio.350

[23] Yeo KJ, Kim EH, Hwang E, Han YH, Eo Y, et al (2013) pH-dependent structural change of the extracellular sensor domain of the DraK histidine kinase from *Streptomyces coelicolor* . Biochem Biophys Res Commun 431: 554–559. 23321311 10.1016/j.bbrc.2013.01.018

[24] Kim YJ, Song JY, Moon MH, Smith CP, Hong SK, et al (2007) pH shock induces overexpression of regulatory and biosynthetic genes for actinorhodin productionin *Streptomyces coelicolor* A3(2). Appl Microbiol Biotechnol 76: 1119–1130. 17609941 10.1007/s00253-007-1083-9

[25] Grzesiek S, Bax A (1992) Improved 3D triple-resonance NMR techniques applied to a 31 kDa protein. . J Magn Reson 96: 432–440.

[26] Clubb RT, Thanabal V, Wagner G (1992) A constant-time three-dimensional triple-resonance pulse scheme to correlate intraresidue 1HN, 15N, and 13C′chemical shifts in 15N, 13C-labelled proteins. J Magn Reson 97: 213–217.

[27] Wittekind M, Mueller L (1993) HNCACB, a high-Sensitivity 3D NMR experiment to correlate amide-proton and nitrogen resonances with the alpha- and beta-carbon resonances in proteins. J Magn Reson 101: 201–205.

[28] Grzesiek S, Bax A (1993) Amino acid type determination in the sequential assignment procedure of uniformly ^13^C/^15^N-enriched proteins. J Biomol NMR 3: 185–204. 8477186 10.1007/BF00178261

[29] Muhandiram DR, Kay LE (1994) Gradient-enhanced triple-resonance three-dimensional NMR experiments with improved sensitivity. J Magn Reson 103: 203–216.

[30] Kay LE, Xu GY, Singer AU, Muhandiram DR, Formankay JD (1993) A gradient-enhanced HCCH-TOCSY experiment for recording side-chain ^1^H and ^13^C correlations in H_2_O samples of proteins. J Magn Reson 101: 333–337.

[31] Guntert P, Mumenthaler C, Wuthrich K (1997) Torsion angle dynamics for NMR structure calculation with the new program DYANA. J Mol Biol 273: 283–298. 9367762 10.1006/jmbi.1997.1284

[32] Herrmann T, Guntert P, Wuthrich K (2002) Protein NMR structure determination with automated NOE assignment using the new software CANDID and the torsion angle dynamics algorithm DYANA. J Mol Biol 319: 209–227. 12051947 10.1016/s0022-2836(02)00241-3

[33] Case DA, Cheatham TE 3rd, Darden T, Gohlke H, Luo R, et al (2005) The Amber biomolecular simulation programs. J Comput Chem 26: 1668–1688. 16200636 10.1002/jcc.20290 PMC1989667

[34] Zweckstetter M, Bax A (2000) Prediction of sterically induced alignment in a dilute liquid crystalline phase: aid to protein structure determination by NMR. J Am Chem Soc 122: 3791–3792.

[35] Mongan J, Simmerling C, McCammon JA, Case DA, Onufriev A (2007) Generalized Born model with a simple, robust molecular volume correction. J Chem Theory Comput 3: 156–169. 21072141 10.1021/ct600085e PMC2975579

[36] Montelione GT, Nilges M, Bax A, Guntert P, Herrmann T, et al (2013) Recommendations of the wwPDB NMR Validation Task Force. Structure 21: 1563–1570. 24010715 10.1016/j.str.2013.07.021 PMC3884077

[37] Shrager RI, Cohen JS, Heller SR, Sachs DH, Schechter AN (1972) Mathematical models for interacting groups in nuclear magnetic resonance titration curves. Biochemistry 11: 541–547. 5011963 10.1021/bi00754a010

[38] Hong YS, Lee D, Kim W, Jeong JK, Kim CG, et al (2004) Inactivation of the carbamoyltransferase gene refines post-polyketide synthase modification steps in the biosynthesis of the antitumor agent geldanamycin. J Am Chem Soc 126: 11142–11143. 15355082 10.1021/ja047769m

[39] Cheung J, Hendrickson WA (2010) Sensor domains of two-component regulatory systems. Curr Opin Microbiol 13: 116–123. 20223701 10.1016/j.mib.2010.01.016 PMC3078554

[40] Zhang Z, Hendrickson WA (2010) Structural characterization of the predominant family of histidine kinase sensor domains. J Mol Biol 400: 335–353. 20435045 10.1016/j.jmb.2010.04.049 PMC3461309

[41] Taylor BL, Zhulin IB (1999) PAS domains: internal sensors of oxygen, redox potential, and light. Microbiol Mol Biol Rev 63: 479–506. 10357859 10.1128/mmbr.63.2.479-506.1999 PMC98974

[42] Moglich A, Ayers RA, Moffat K (2009) Structure and signaling mechanism of Per-ARNT-Sim domains. Structure 17: 1282–1294. 19836329 10.1016/j.str.2009.08.011 PMC3092527

[43] Davoodi J, Wakarchuk WW, Campbell RL, Carey PR, Surewicz WK (1995) Abnormally high pKa of an active-site glutamic acid residue in *Bacillus circulans* xylanase. The role of electrostatic interactions. Eur J Biochem 232: 839–843. 7588724

[44] Inoue M, Yamada H, Hashimoto Y, Yasukochi T, Hamaguchi K, et al (1992) Stabilization of a protein by removal of unfavorable abnormal pKa: substitution of undissociable residue for glutamic acid-35 in chicken lysozyme. Biochemistry 31: 8816–8821. 1390669 10.1021/bi00152a018

[45] Ragona L, Fogolari F, Catalano M, Ugolini R, Zetta L, et al (2003) EF loop conformational change triggers ligand binding in beta-lactoglobulins. J Biol Chem 278: 38840–38846. 12857741 10.1074/jbc.M306269200

