# Supplementary material for: Mechanism of the pH-Induced Conformational Change in the Sensor Domain of the DraK Histidine Kinase via the E83, E105, and E107 Residues
Source: PLoS One. 2014 Sep 9;9(9):e107168. doi: 10.1371/journal.pone.0107168 (PMC4159317; doi:10.1371/journal.pone.0107168)
Supplement: Appendix S1 — Detailed methods and results including NMR and CD spectra. (DOCX) [file pone.0107168.s001.docx]

**Supporting Information: Appendix S1**

**Mechanism of the pH-induced conformational change in the sensor domain of the DraK histidine kinase via the E83, E105, and E107 residues**

**Kwon Joo Yeo^1,†^, Young-Soo Hong^2,†^, Jun-Goo Jee^3^, Jae Kyoung Lee^2^, Hyo Jeong Kim^2^, Jin-Wan Park^1^, Eun-Hee Kim^1^, Eunha Hwang^1^, Sang-Yoon Kim^4^, Eun-Gyeong Lee^4^, Ohsuk Kwon^4,^*, Hae-Kap Cheong^1,^***

**1** Division of Magnetic Resonance, Korea Basic Science Institute (KBSI), Ochang, Chungbuk, Republic of Korea, **2** Chemical Biology Research Center, Korea Research Institute of Bioscience and Biotechnology (KRIBB), Ochang, Chungbuk, Republic of Korea, **3** College of Pharmacy, Kyungpook National University, Kyungpook, Republic of Korea, **4** Biochemicals and Synthetic Biology Research Center, KRIBB, Yuseong-Gu, Daejeon, Republic of Korea

*To whom correspondence should be addressed (E-mail: [haekap@kbsi.re.kr](mailto:haekap@kbsi.re.kr), [oskwon@kribb.re.kr](mailto:oskwon@kribb.re.kr), TEL: +82 43-240-5062, FAX: +82-43-240-5059)

**^†^** These authors contributed equally to this work.

***Construction of draR and draK gene deletion mutants in S. coelicolor.***

For *draR* (SCO3063) disruption, approximately 1.36 kb of the 5’ region of the *draR* gene was amplified from the genomic DNA of *S. coelicolor* by PCR using a forward primer with an *EcoR*I site added and a reverse primer with a *Pst*I site added (Table S1). The PCR product was cloned into the pCR-TOPO2.1 vector to produce pTA-314. Approximately 1.2 kb of the 3’ region of the *draR* gene was also amplified by PCR using a primer set adding *Kpn*I and *Hind*III sites to its 5’ and 3’ ends, respectively. The PCR product was cloned into the pCR-TOPO2.1 vector to produce pTA-324. The pTA-314 plasmid was digested with *EcoR*I and *Pst*I, and the pTA-324 plasmid was digested with *Kpn*I and *Hind*III to isolate DNA fragments corresponding to the 5’- and 3’- flanking regions of *draR,* respectively. The 1.1 kb *aph*II gene (kanamycin resistance gene; KanR) was obtained from a *Pst*I, *Kpn*I-digested pFD-neoS plasmid [1]. All three fragments were ligated together into an *EcoR*I-*Hind*III digested pKC1139 vector to produce pKC-3063A [2]. The construct was inserted into *S. coelicolor* by conjugation with *E. coli* ET12567(pUZ8002). For *draK* (SCO3062) disruption, approximately 1.37 kb of the 5’ region of *draK* gene was amplified from the genomic DNA of *S. coelicolor* by PCR using a forward primer with an added *EcoR*I site and a reverse primer with an added *Pst*I site (Table S1). The PCR product was cloned into the pCR-TOPO2.1 vector to produce pTA-214. Approximately 1.58 kb of the 3’ region of *draK* was also amplified by PCR using a primer set adding *Kpn*I and *Hind*III sites to its 5’ and 3’ ends, respectively. The PCR product obtained was cloned into the pCR-TOPO2.1 vector to produce pTA-224. The pTA-214 plasmid was digested with *EcoR*I and *Pst*I, and the pTA-224 plasmid was digested with *Kpn*I and *Hind*III to isolate DNA fragments corresponding to the 5’- and 3’- flanking regions of *draK,* respectively. The 1.1 kb *aph*II gene was obtained from a *Pst*I, *Kpn*I digested pFD-neoS plasmid [1]. All three fragments were ligated together into an *EcoR*I-*Hind*III digested pKC1139 vector to create pKC-3062B. The construct was inserted into *S. coelicolor* by conjugation with *E. coli* ET12567 (pUZ8002) [2]. Intergeneric conjugation between *E. coli* and *Streptomyces* was performed as previously described [2] with minor modifications. Transformants resistant to apramycin and kanamycin were selected and grown in fresh R2YE/kanamycin liquid medium at 37℃ for 4 days to force the integration of disruption cassette DNA from gene disruption vectors into chromosomal DNA. The resulting gene disruption mutants (Δ*draK* and Δ*draR*) were selected on R2YE/kanamycin medium and confirmed by PCR with the relevant primer sets (Table S1) using total genomic DNA from each mutant as template. PCR primers were designed around *draR* (SCO3063) and *draK* (SCO3062) with primers binding 5’ of the *draR* (SCO3063) and *draK* (SCO3062) gene regions for sense primers, and a sense primer region downstream was used for antisense (Table S1). As a result, 1.2 kb of the PCR product was detected for wild type and Δ*draR*, and a 2.3 kb PCR product was shown for Δ*draK*. This result demonstrated insertion of the *aph*II gene (1 kb) in the middle of the *draK* gene. A 1.4 kb PCR product was detected for the wild type and Δ*draK* genes, and a 2.5 kb PCR product was observed for Δ*draR*. This result demonstrated insertion of the *aph*II gene (1 kb) in the middle of the *draR* gene. In addition, PCR products with the C63-F and Neo-R primer set were detected for Δ*draK* (2.4 kb) and Δ*draR* (1.5 kb), and a PCR product was not observed for the wild type. This result demonstrated the insertion of the *aph*II gene (1 kb) in the *draK* and *draR* genes (Figure S2).

**Table S1**. Primers used in this study.

| Target | Name | Sequences (5’-3’) | Restriction  Enzyme site |
| --- | --- | --- | --- |
| *draR*  (SCO3063) | 63-1E | gaaTTCTCGCCGATGGTGCCGATGGTCAGCGG | *EcoR*I |
|  | 63-2P | ctGCAGCTCGGCGAGGCGGAAGGGCTTGGTG | *Pst*I |
|  | 63-3K | ggtACCGGGCCTGGATGGGAGAGGAGGAGCTC | *Kpn*I |
|  | 63-4H | aaGCTTGCCGGAGCTGACGATGGCCCGCCCC | *Hind*III |
| *draK*  (SCO3062) | 62-1E | gaaTTCCCCCAGGATGGCACCCCACAGCCGCA | *EcoR*I |
|  | 62-2P | ctgCAGCTCGGGGACGCCGTACCGCTTGTGCC | *Pst*I |
|  | 62-3K | ggtACCGGGTCGCGGACGTGCTGGACTCCTCC | *Kpn*I |
|  | 62-4H | GAAGCTTGAACCTGATGCCGAGCGGaagctt | *Hind*III |
| Mutant confirmation | C63-F | gctgccgatcaggaactggaggacg |  |
|  | Neo-R | CGCATCGCCTTCTATCGCCTT CTTG |  |

**Figure S1**. Inactivation of the *draR* (A) and *draK* (B) genes in *S. coelicolor*. *Kan^R^*, kanamycin resistance gene; *apr^R^*, apramycin resistance gene.

**A)**


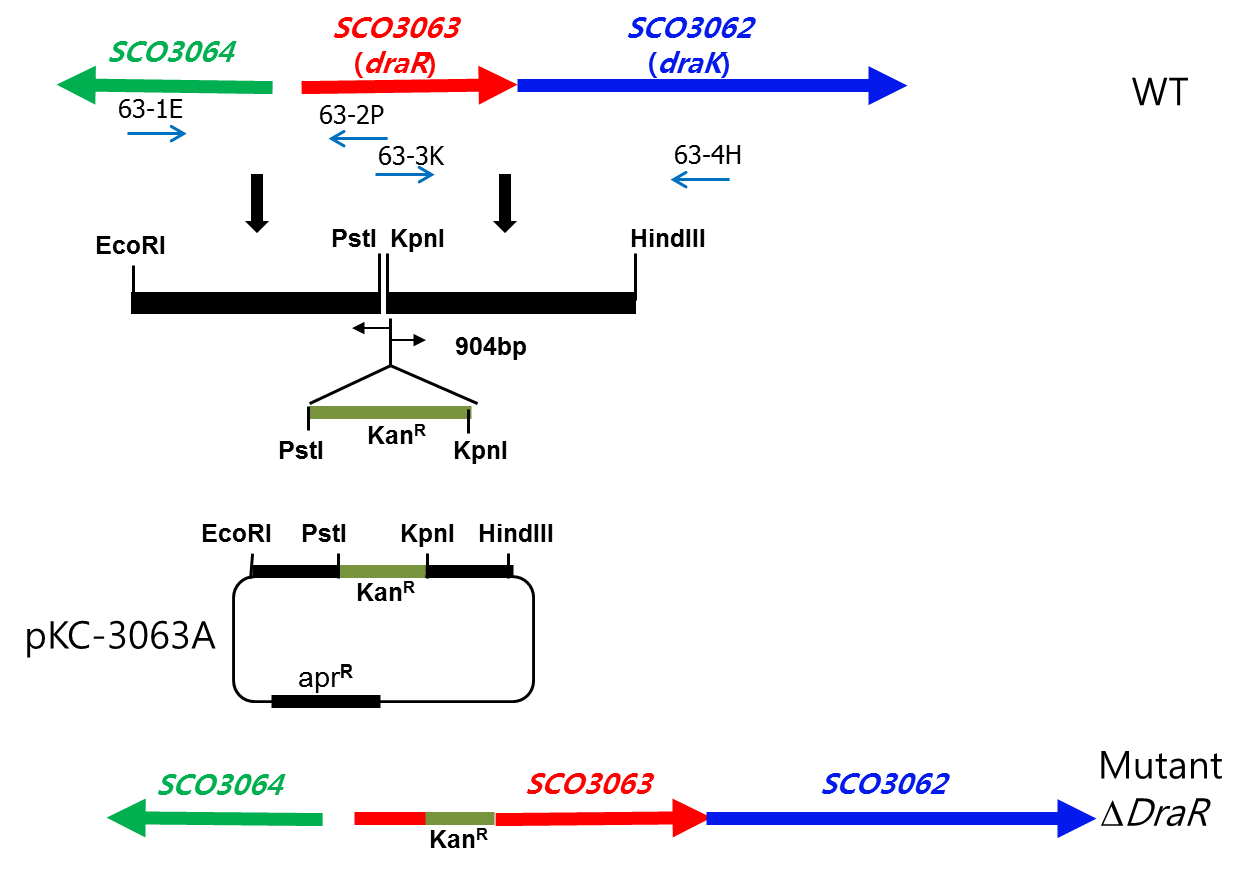


**B)**


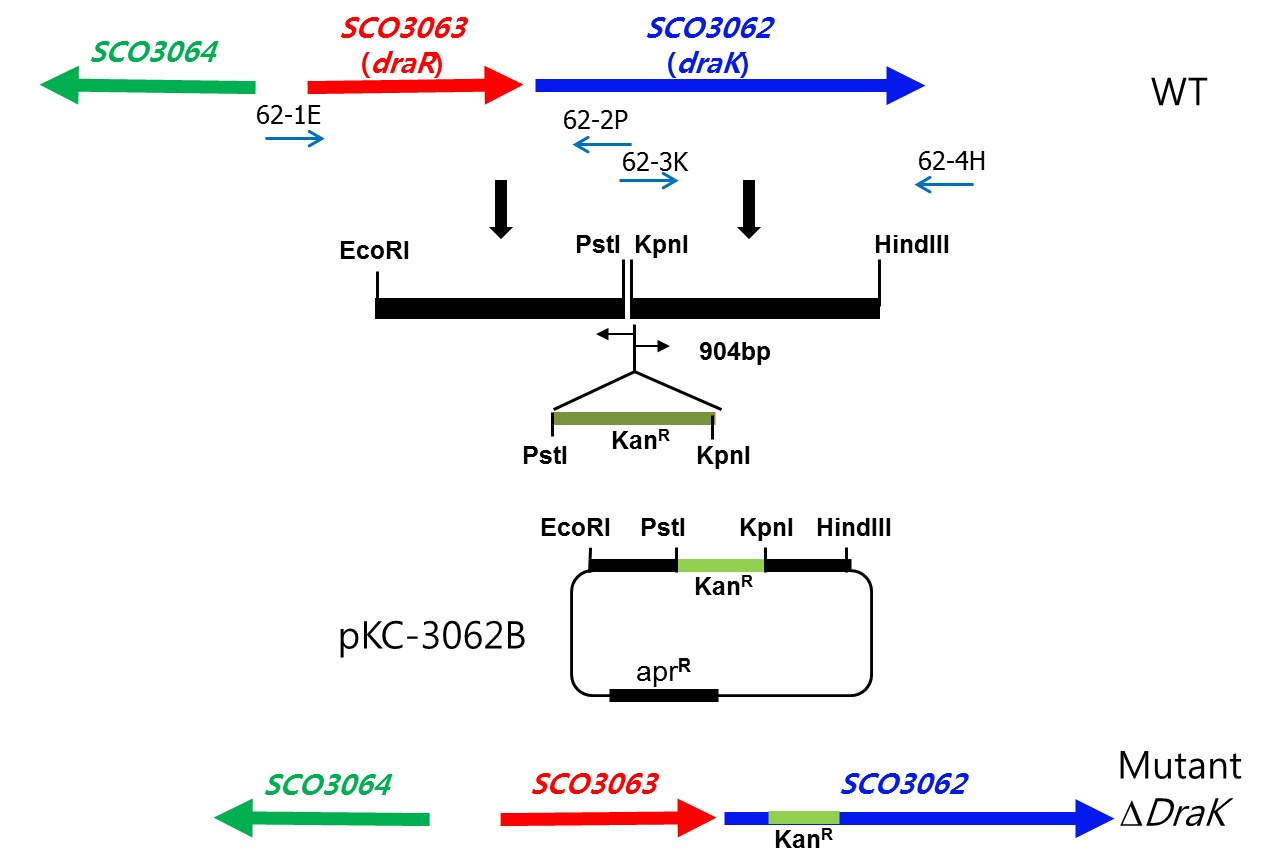


**Figure S2**. Confirmation of insertional gene inactivation by PCR using the total genomic DNA of each mutant as template (A). M, 1 kb ladder. The relevant primers used to amplify the desired DNA fragments are indicated (B).

A)


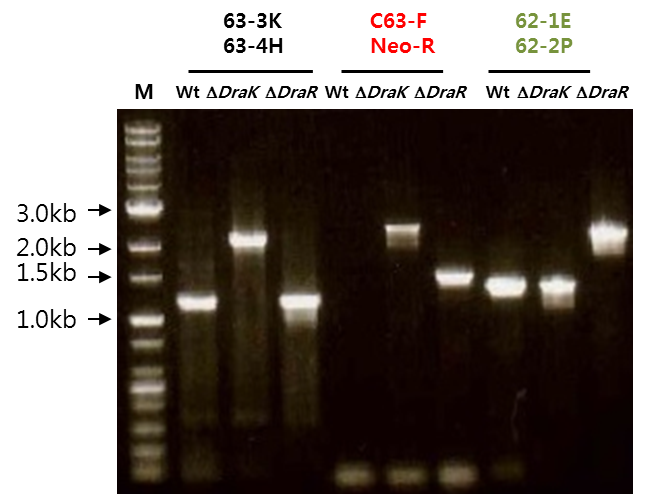


B)


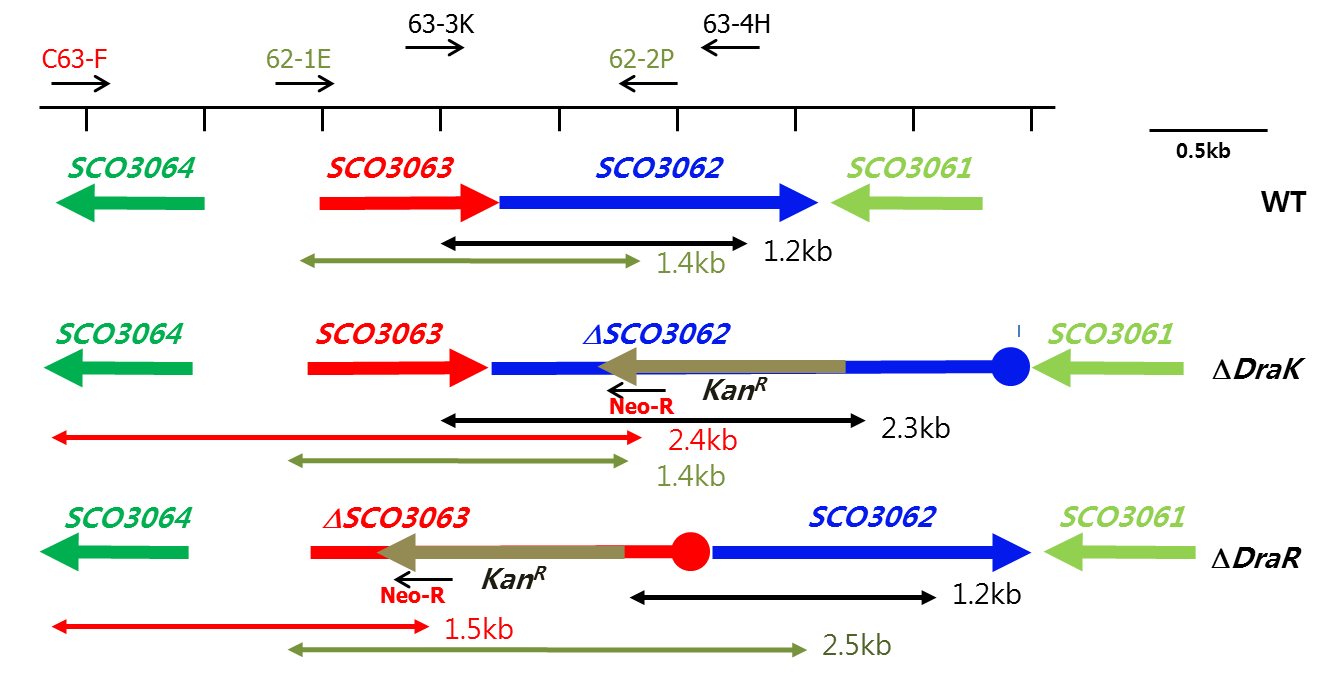


**Figure S3**. ^1^H-^15^N HQSC spectrum and backbone assignment for the ESD (E83Q) mutant at pH 4.5 (A). ^1^H-^15^N HQSC spectra for the wild type (red) and the E83Q mutant (blue) (B).

A)


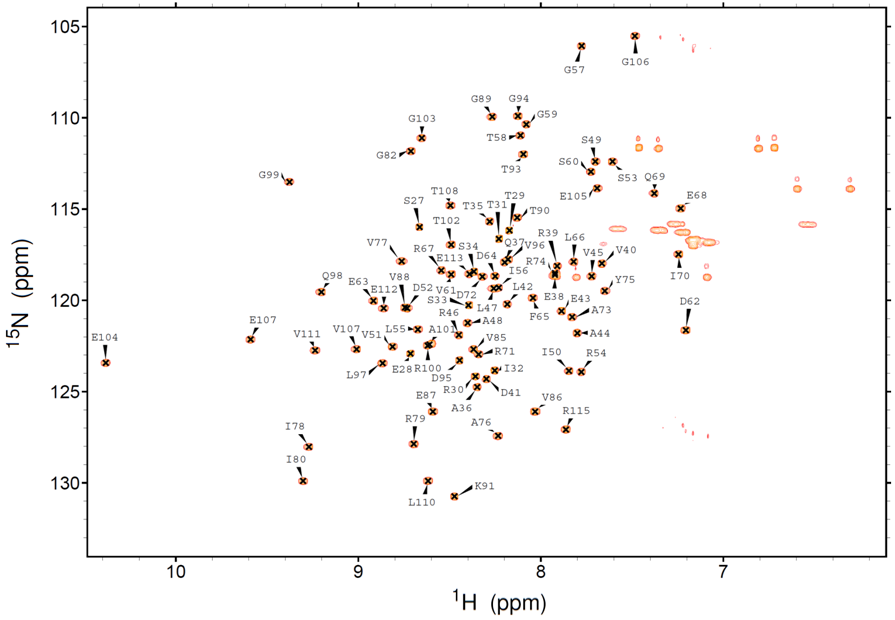


B)

**Table S2**. NMR restraints and statistics for the ensemble of the 20 lowest energy structures calculated for the DraK ESD.

Average AMBER energies (kcal/mol)

AMBER -4,212

Constraint 10

Completeness of resonance assignments (%) 99.8

Conformation restricting restraints

Distance restraints 1,750

Intra (|i-j|=0) 398

Sequential (|i-j|=1) 488

Medium range (1<|i-j|<5) 390

Long range (|i-j|>4) 474

No. of restraints per residue 19.4

No. of long-range restraints per residue 5.3

Residual restraints violations

Average no. of distance violation (> 0.5 Å) 0.0 (0.31 Å max)

Average no. of angle violation (> 5.0°) 0.0 (1.40 ° max)

Residual dipolar coupling restraints

No. of restraints 82

Average deviation (Hz) 0.81

Average R-factor/Q-factor 0.993/0.073

Model quality

Rmsd backbone atoms (Å) (11-88 residues) 0.29

Rmsd heavy atoms (Å) (11-88 residues) 0.78

Rmsd bond lengths (Å) 0.011

Rmsd bond angles (°) 2.1

MolProbity Ramachandran statistics

Most favored regions (%) 88.1

Allowed regions (%) 11.9

Disallowed regions (%) 0.0

Global quality scores

Verify3D 0.28

ProsaII (Z-score) -5.5

MolProbity clash score 0.69

Model contents

Ordered residue ranges 11–88

Total no. of residues 90

PDB ID code 2MJ6

**Figure S4.** The change in the CD signal intensity of the E83D mutant at 218 nm over a pH range of 4.2-7.4. The midpoint of the transition occurs at approximately pH 5.7.

**Figure S5**. ^1^H-^15^N HQSC spectrum for the ESD (E83L/E105L/E107A) mutant at pH 4.5 (A) and 7.5 (B)

**Figure S6**. CD spectra for the ESD (E83L/E105L/E107A) mutant at pHs 4.5, 7.5 and 10.0

**References**

1. Denis F, Brzezinski R (1991) An improved aminoglycoside resistance gene cassette for use in gram-negative bacteria and Streptomyces. FEMS Microbiol Lett 65: 261-264.

2. Bierman M, Logan R, O'Brien K, Seno ET, Rao RN, et al. (1992) Plasmid cloning vectors for the conjugal transfer of DNA from Escherichia coli to Streptomyces spp. Gene 116: 43-49.
